# Facile hydrothermal synthesis of ternary Ni–Co–Se/carbon nanotube nanocomposites as advanced electrodes for lithium storage†

**DOI:** 10.1039/c8ra05142a

**Published:** 2018-08-13

**Authors:** Jingguo Ding, Hui Xu, Xiaobo Chen

**Affiliations:** School of New Energy and Electronic Engineering, Yancheng Teachers University Yancheng 224051 PR China chenxbok@126.com +86-515-88233177

## Abstract

Ternary Ni–Co–Se/carbon nanotube nanocomposites have been successfully prepared *via* a one-step hydrothermal strategy. When used as electrode materials for lithium-ion batteries, the Ni–Co–Se/CNT composite exhibits good lithium storage performances including excellent cycling stability and outstanding specific capacity, good cycling stability, and high initial coulombic efficiency. A high specific capacity of 687.8 mA h g^−1^ after 100 charge–discharge cycles at a current density of 0.5 A g^−1^ with high cycling stability is achieved. The excellent battery performance of Ni–Co–Se/CNT should be attributed to the synergistic effect of Ni and Co ions and the formed network structure.

## Abstract 2

A Ni–Co–Se/CNT composite exhibits outstanding Li-ion storage performance with respect to high reversible Li-storage capacity, high cyclability and high rate performance.

## Graphical abstract

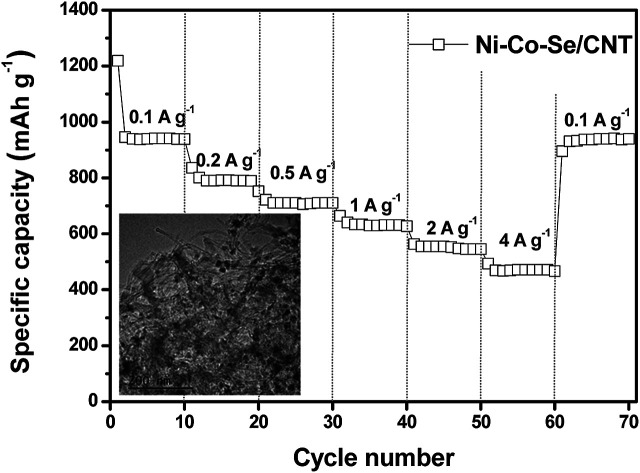

## 1. Introduction

Lithium-ion batteries (LIBs) have been widely used as the power source for portable electronic devices such as mobile phones and laptops owing to their high energy density and long cycle life.^1–5^ At present, graphite is widely used as the anode material for LIBs. However, it is difficult for the graphite electrode to meet the high energy density and portability requirements of new generation LIBs due to its relatively low specific capacity (372 mA h g^−1^).^6,7^ Therefore, it is challenging, but desirable, to find a new attractive material as an anode with higher energy density.

As an available anode materials, low-cost transition-metal chalcogenides (TMCs) can in principle give much higher lithium-storage capacity due to their unique physical, chemical, and electronic properties.^8–11^ However, relatively low electrical conductivity and huge volume change during the charge–discharge process are major drawbacks that limit their utility for energy storage.^8,10^ Thus the electrochemical performance of these compounds has to be improved *via* both structural and compositional engineering for real applications.^10–13^ A viable solution is preparing the composites consisting of TMCs and carbonaceous materials (such as amorphous carbon,^14,15^ graphene,^16,17^ carbon nanotubes^18,19^) to improve conductivity of electrodes. The carbonaceous materials are introduced to improve the electrical conductivity and enhance the electrochemical performance of the TMCs anodes.^14,16,18^ Among the carbonaceous materials, carbon nanotubes (CNT) have been recognized as a promising candidate for used as the anodes of LIBs^20–22^ due to their benefits including high electrical conductivity, chemical stability and large specific surface area.^23^

Herein, the well dispersed Ni–Co–Se/CNT composite anode material for LIBs has been prepared *via* simple one-step hydrothermal method. The as-prepared Ni–Co–Se/CNT composite exhibits higher capacity and better cycle stability compared to CoSe_2_/CNT or NiSe_2_/CNT, and is excellent compared with those of many previous related reports.

## 2. Experimental details

### 2.1. Material synthesis

In this work, CNT are purchased from Tanfeng Tech. Inc (Suzhou, China). Other reagents are analytical grade without further purification and purchased from Shanghai Zhanyun Chemical Co., Ltd., Shanghai, China. Ni–Co–Se/CNT composites were prepared by a facile hydrothermal method. Firstly, 5 mg CNT were dispersed in 20 mL of ethylene glycol to form a dispersion through ultrasonication (solution A). Then, 0.757 g of Co(NO_3_)_2_·6H_2_O, 0.378 g of Ni(NO_3_)_2_·6H_2_O (*n*_Ni_ : *n*_Co_ = 1 : 2) and 2.6 mL of ethylenediamine were dissolved in 40 mL distilled water (solution B). Then 0.28 g SeO_2_ (*n*_Ni_ : *n*_Co_ : *n*_Se_ = 1 : 2 : 4) and 2 mL hydrazine hydrate were dissolved in 20 mL distilled water (solution C). Solution A, B and C were mixed under stirring for 20 min, and the reaction mixture was transferred to a Teflon lined stainless steel autoclave. The hydrothermal reaction was set at 180 °C for 24 h. After the autoclave cooled down to room temperature, the as-obtained product was collected by centrifugation and washed for several times using deionized water and absolute ethanol, and then dried at 80 °C overnight in a vacuum oven. For comparison, CoSe_2_/CNT and NiSe_2_/CNT were synthesized through the same procedure except that only 0.757 g Co(NO_3_)_2_·6H_2_O or 0.378 g of Ni(NO_3_)_2_·6H_2_O was added.

### 2.2. Characterization and electrochemical measurements

The X-ray diffraction (XRD) patterns were recorded in an XRD diffractometer (X'pert MPD Pro, Philips, Netherlands) using Cu Kα radiation (*λ* = 1.5418 Å). The morphologies of the products were examined by a scanning electron microscopy (SEM, Zeiss Supra 35VP, Berlin, Germany) with an energy-dispersive X-ray spectrometer (EDS). Transmission electron microscopy (TEM) images were recorded by JEOL JEM 2100 TEM operated at 200 kV. The surface composition and chemical states of the products were determined by the X-ray photoelectron spectroscopy (XPS) using a Thermo-ESCALAB 250XI (Thermo, USA) instrument with non-monochromated Al Kα 1486.6 eV radiation. All the binding energies were calibrated by the reference C 1s peak at 284.6 eV.

The electrochemical measurements were detected on a Land CT2001A multi-channel battery testing instrument by using CR2025 coin-type cells. The working electrode prepared by mixing active material, acetylene black and sodium alginate (SA) binder in a weight ratio of 80 : 10 : 10 using deionized water as solvent to form well-dispersed slurry. Then, the slurry was spread onto a pure copper foil, and dried at 60 °C overnight, then the electrode was cut into pellets with a diameter of 1.0 cm and dried for another 12 h in a vacuum oven at 60 °C. The mass loading of active material was about 2.0 mg. The coin cells were assembled in an argon-filled glove box with lithium foil as the counter electrode. The electrolyte system chosen for LIBs was 1 M LiPF_6_ (Aldrich) in ethylene carbonate/dimethyl carbonate/diethyl carbonate (EC : DMC : DEC = 1 : 1 : 1 in volume). Cyclic voltammetry (CV) measurements were carried out on a CHI660C electrochemical workstation in a voltage range of 0.01–3.0 V *vs.* Li/Li^+^ at a scan rate of 0.1 mV s^−1^. All the tests were performed at constant temperature (25 °C).

## 3. Results and discussions

### 3.1. Morphology and composition

**Fig. 1:**
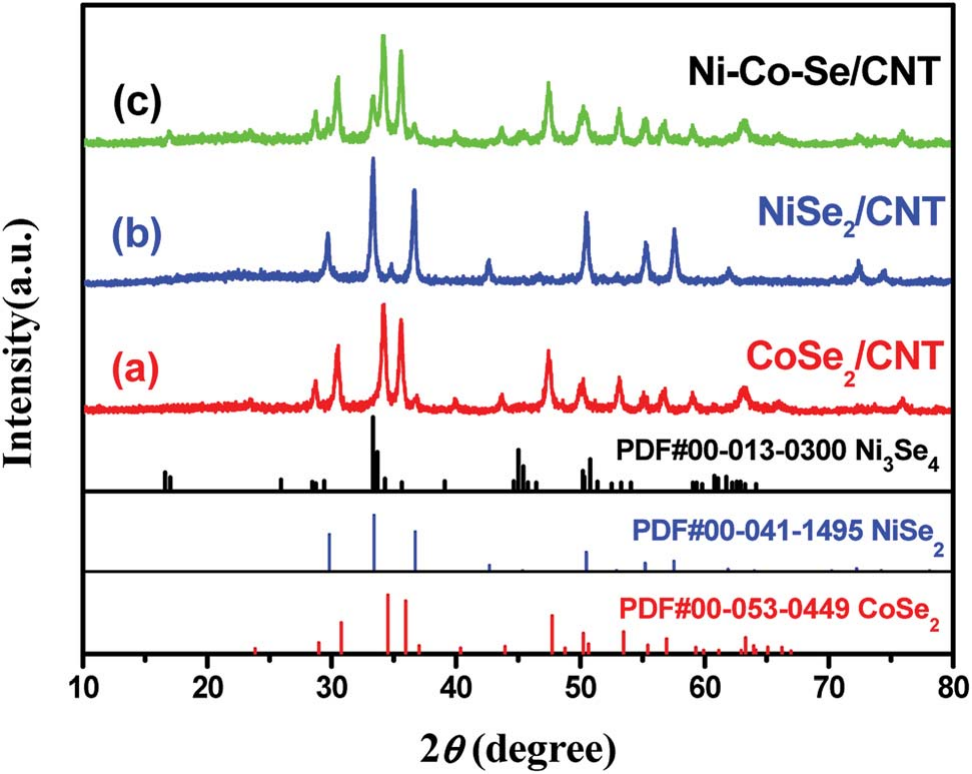
XRD patterns of the samples (a) CoSe_2_/CNT, (b) NiSe_2_/CNT, and (c) Ni–Co–Se/CNT.

Fig. 1 shows the typical XRD patterns of the samples. CNT peaks were not observed in the XRD patterns of composites as the resolution of XRD analysis is almost 5 wt%.^24^ Direct evidence to the existence of CNT will be given in the SEM and TEM observation section. In Fig. 1(a), the typical diffraction peaks of orthorhombic CoSe_2_ can be found, which can be indexed as the standard data (ICDD PDF no. 00-053-0449). The diffraction peaks given in Fig. 1(b) can be indexed to the standard pattern for cubic NiSe_2_ (ICDD PDF no. 00-041-1495). The diffraction peaks of the Ni–Co–Se (Fig. 1(c)) can be excellently assigned to the orthorhombic CoSe_2_ (ICDD PDF no. 00-053-0449)^25^ and a small fraction of cubic Ni_3_Se_4_ (ICDD PDF no. 00-013-0300).^26^ With introducing of Co ions, the peaks assigned to NiSe_2_ disappear. The results reveal that Ni and Co ions have strong chemical interaction and they tend to form Ni_3_Se_4_ phase. Also, it is obvious that there are no other peaks in the patterns indicates that the synthesized products are of pure quality. Several well-defined peaks appear at 23.8°,28.7°, 30.5°, 34.3°, 35.6°, 36.9°, 40.1°, 43.7°, 47.5°, 50.3°, 53.2°and 63.1° corresponding to orthorhombic CoSe_2_ (110), (011), (101), (111), (120), (200), (210), (121), (211) (002), (031) and (122), respectively. The peaks at 17.0°, 33.6°, 45.3°, 50.1° and 53.2° corresponding to (002) (202), (204) (020) and (002) crystal planes of Ni_3_Se_4_, respectively, are observed. The XRD pattern of the synthesized Ni–Co–Se consists of orthorhombic CoSe_2_ and monoclinic Ni_3_Se_4_.

**Fig. 2:**
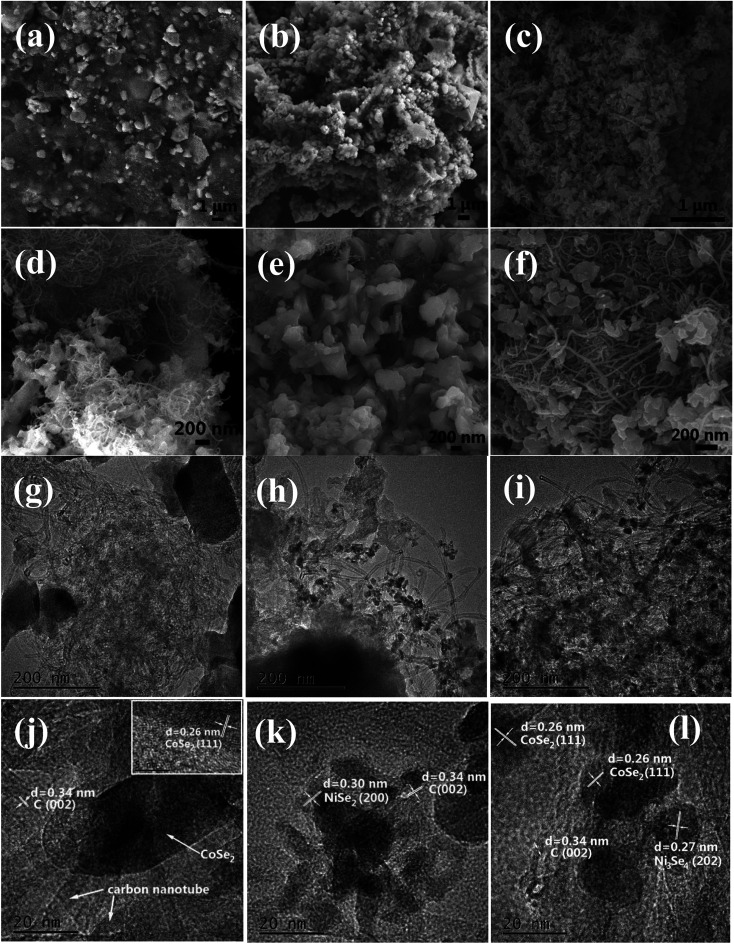
SEM images of CoSe_2_/CNT (a and d), NiSe_2_/CNT (b and e) and Ni–Co–Se/CNT (c and f). TEM and HRTEM images of CoSe_2_/CNT (g and j), NiSe_2_/CNT (h and k) and Ni–Co–Se/CNT (i and l).

Fig. 2(a–f) show the representative SEM images and Fig. 2(g–l) show the TEM and HRTEM images of CoSe_2_/CNT, NiSe_2_/CNT, and Ni–Co–Se/CNT. And the crystalline nature was confirmed by the HRTEM. It can be seen that all the samples exhibit similar irregular particle-like morphologies, and the particles connected with each other to some degree. Fig. 2(i and l) exhibited the TEM images of Ni–Co–Se nanoparticles which were distributed uniformly and continuously onto the CNT surface with an average diameter of 15 nm. Moreover, compared with the CoSe_2_/CNT and NiSe_2_/CNT, the loading capacity of Ni–Co–Se was significantly increased and the particles were deposited in more uniform structures.

**Fig. 3:**
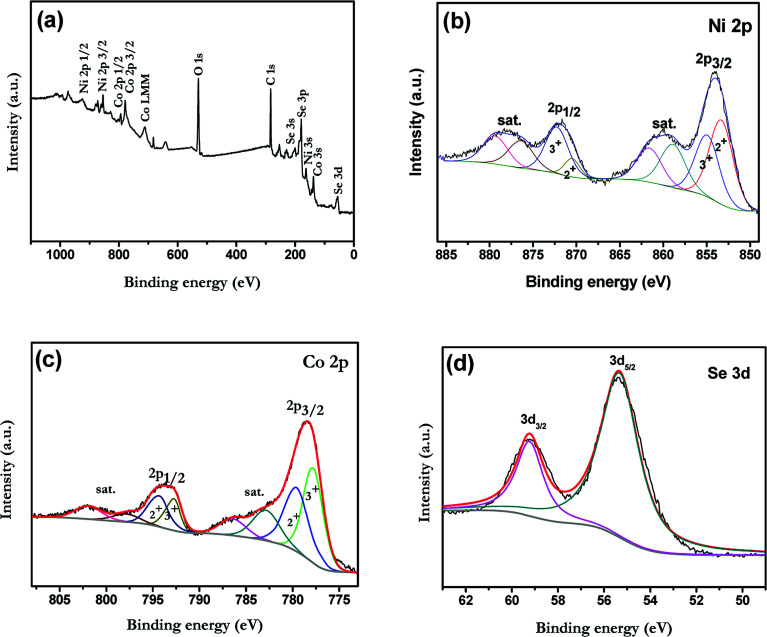
(a) Wide scan XPS survey and high resolution XPS spectra of Ni–Co–Se/CNT for (b) Ni 2p, (c) Co 2p, (d) Se 3d.

The elemental composition and their chemical states in the near surface range of samples were analyzed by XPS. The results of Ni–Co–Se/CNT are shown in Fig. 3 and those of CoSe_2_ and NiSe_2_ are given in Fig. S2.† Peaks in the survey spectrum of the Ni–Co–Se/CNT are ascribed to the elements of Ni, Co, Se, C, and O. The O 1s peak in the survey spectrum of Ni–Co–Se/CNT can be assigned to adsorb H_2_O on the surface. Results show that Ni–Co–Se/CNT consists of Ni, Co, Se and C elements on the near-surface of product, which is in consistent with our experiment. Fig. 3(b–d) show the XPS spectra of Ni 2p, Co 2p and Se 3d, respectively. High-solution spectra of Ni 2p and Co 2p of all samples can be divided into multipeaks, including spin–orbit doublets and shakeup satellites by Gaussian fitting method. Fig. 3(b) shows the Ni 2p spectrum of Ni–Co–Se/CNT, the fitting peaks at 853.4 and 870.5 eV are assigned to Ni^2+^, and the peaks at 855.1 and 872.4 eV are ascribed to Ni^3+^.^27,28^ For the Co 2p spectrum, the first doublet (at 777.9 and 792.9 eV) and the second doublet (at 779.7 and 795.0 eV) are characteristics of Co^3+^ and Co^2+^.^29,30^ Moreover, as shown in Fig. 3(d), Se 3d peaks are divided into two peaks. The peaks located at 59.2 eV and 55.3 eV are attributed to the Se 3d_3/2_ and 3d_5/2_, which are the typical peaks of metal–selenium bonds.^31^ As shown in Fig. S1,† the Co 2p spectrum of CoSe_2_ and Ni 2p spectrum of NiSe_2_ are also can be well fitted with two spin–orbit doublets and two shake-up satellites, which are similar to those of Ni–Co–Se/CNT. The XPS analysis results are consistent with the XRD measurement.

**Fig. 4:**
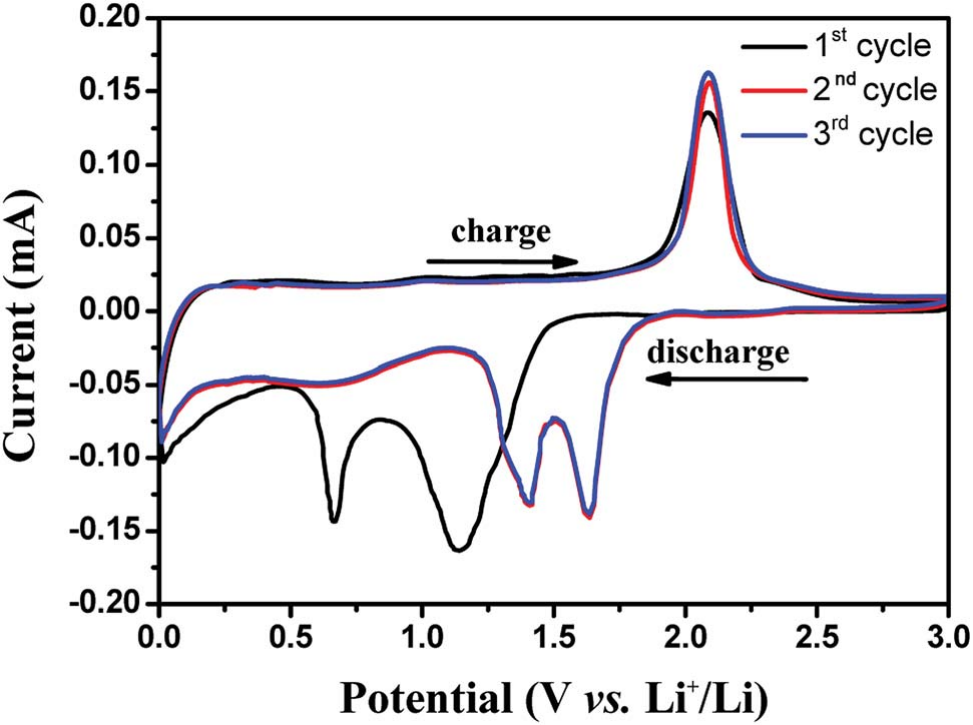
Cyclic voltammogram profiles of the Ni–Co–Se/CNT composite with the scan rate of 0.1 mV s^−1^.

The electrochemical properties of the as-synthesised Ni–Co–Se/CNT, CoSe_2_/CNT and NiSe_2_/CNT for lithium storage are evaluated using coin-type cell. CV measurements are carried out to analyze the electrochemical behavior of the electrodes. The representative CV curves for the first three cycles in the potential range between 0 and 3 V at a scan rate of 0.1 mV s^−1^ are shown in Fig. 4 and Fig. S2.† As shown in Fig. 4, three reversible redox peaks exist in LIBs, indicating a multiphase reaction mechanism during the electrochemical process. The first cycle of CV curves is different from the others, suggesting an irreversible reaction occurs in the first discharge process. In details, two reduction peaks appear at approximately 0.66 V and 1.14 V, and one oxidation peak at 2.1 V appear in the first cathodic/anodic process. The peaks at 0.66 V and 1.14 V corresponds to the reduction of Ni–Co–Se to metallic Ni and Co nanocrystals dispersed into the Li_2_Se matrix.^15,32,33^ In the subsequent cycles, the cathode peaks of Ni–Co–Se shift to higher potential of 1.40 and 1.60 V, the anodic peaks almost remain unchanged. From the second cycle onwards, the CV curves are almost overlapped, indicating the excellent electrochemical reversibility of Ni–Co–Se electrode. Similar features are also observed in the CV curves of CoSe and NiSe.

**Fig. 5:**
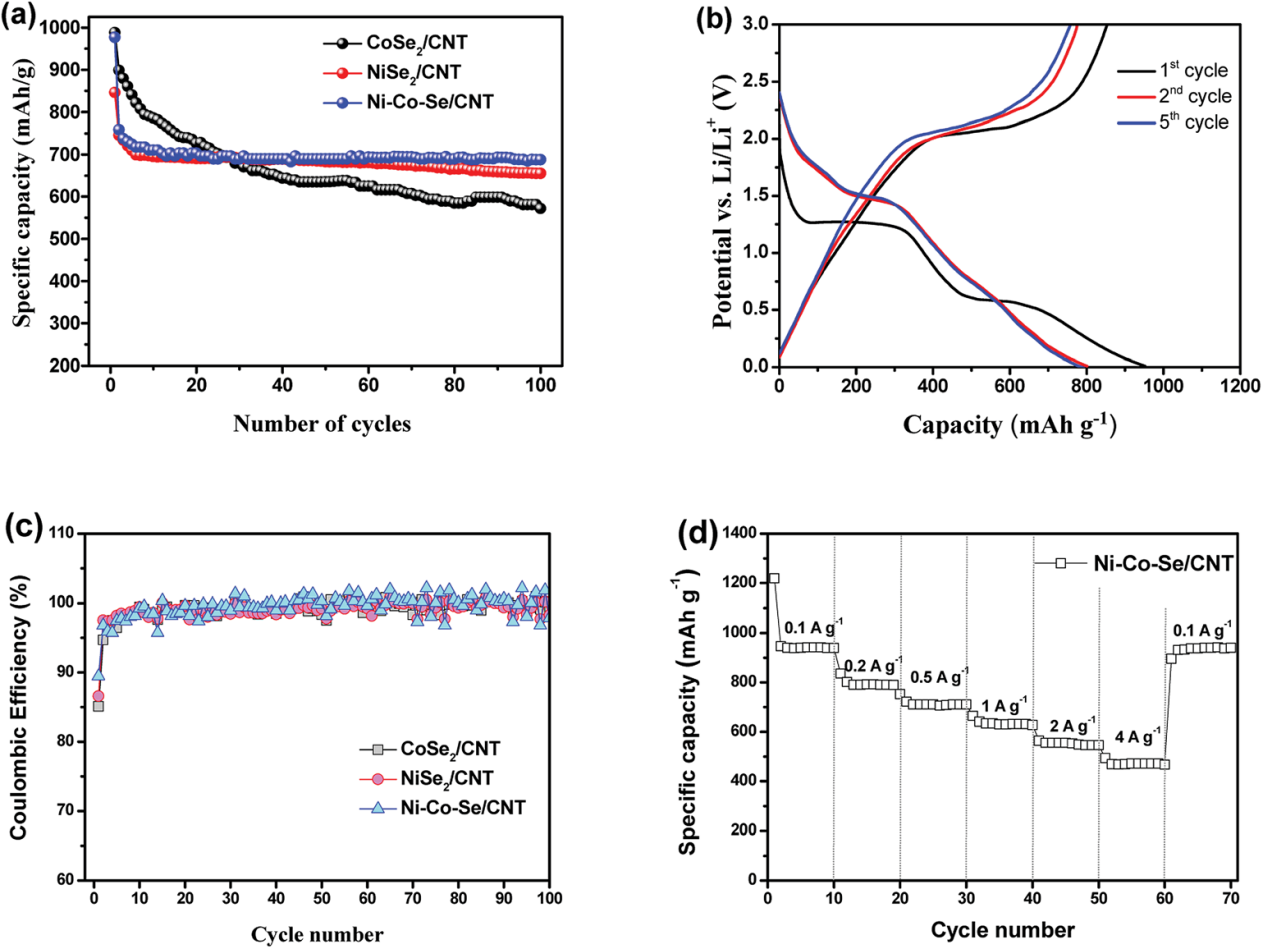
(a) Comparison of the cycling performances of the CoSe_2_/CNT, NiSe_2_/CNT and Ni–Co–Se/CNT during 100 cycles at a current density of 0.5 A g^−1^. (b) Galvanostatic discharge/charge voltage profiles of the Ni–Co–Se/CNT composite at a current density of 0.2 A g^−1^. (c) Coulombic efficiency at a current density of 0.2 A g^−1^. (d) Rate performance of Ni–Co–Se/CNT.

Long cycle life is a crucial parameter for LIBs electrode materials, and the cycling performances of the samples are measured at 0.5 A g^−1^. As shown in Fig. 5(a), in the initial 23 cycles, the specific capacities of all samples decline with the increase of cycle numbers. The specific capacities of CoSe_2_ decrease more slowly than that of NiSe_2_. But after 23 charge/discharge cycles, the sample of NiSe_2_ present higher specific capacity and more stable cycle performance. Look from whole, the sample of Ni–Co–Se/CNT has a more stable cycle performance and higher specific capacity. The results suggest that Co ion are helpful for improving its initial capacity and Ni ion are beneficial for improving the cycling stability of Ni–Co–Se/CNT. Benefit from the synergistic effect of Ni and Co ions and its unique structure, Ni–Co–Se/CNT has the best cycling stability after 100 charge and discharge cycles. Most notably, the capacity and cycling stability of Ni–Co–Se/CNT composite are outstanding in most of the reported transition-metal chalcogenide based anode materials (Table S1†). Due to the best electrochemical performance of long cycle life and high specific capacity, then the Ni–Co–Se/CNT was selected for further study below.

Fig. 5(b) shows the charge–discharge voltage profiles of Ni–Co–Se/CNT electrodes for 1^st^, 2^nd^ and 5^th^ cycles at a current density of 0.2 A g^−1^, separately. There is a potential plateau at ∼1.27 and ∼0.59 V in first discharge process and from the second cycle onward, the discharge plateaus changed to 1.49 V, and all the charge curves have a similar voltage plateaus at around 2.1 V, indicating similar redox reactions occur. All the voltage plateaus in the discharge and charge curves (Fig. 5(b)) agree well with the cathodic and anodic peaks in the CV curves. The first discharge and charge capacities at a current density of 0.2 A g^−1^ are about 953 and 853 mA h g^−1^, respectively, corresponding to an initial coulombic efficiency of 89.5%. The evolution of coulombic efficiency of all samples at a current density of 0.2 A g^−1^ with cycling is presented in Fig. 5(c). It is found that the initial coulombic efficiencies are 85.1%, 86.6% and 89.5% for the CoSe_2_/CNT, NiSe_2_/CNT and Ni–Co–Se/CNT electrodes, respectively. After initial few cycles, all electrodes exhibit high coulombic efficiency of >95%. Ni–Co–Se/CNT electrode is the best, reaching 97.5% in the second cycle, indicating excellent reversible storage of Li.

The rate performance of the Ni–Co–Se/CNT electrode was also performed, and the results are shown in Fig. 5(d). The electrode was tested at 0.1, 0.2, 0.5, 1 and 2 A g^−1^, and capacities of 940, 792, 706, 629 and 554 mA h g^−1^ can be obtained, respectively. Even at a high current density of 4 A g^−1^, the Ni–Co–Se/CNT electrode still possesses a capacity of 472 mA h g^−1^. In addition, after the current density returns to 0.1 A g^−1^, the specific capacity is recovered to 931 mA h g^−1^ (99% of the 10th cycle reversible capacity). These results indicate that the Ni–Co–Se/CNT electrode can tolerate a very high current rate of 4 A g^−1^ and its high reversible capacity. This excellent rate performance can be ascribed to the superior lithium ion and electron mobility obtained from the distinctive structure advantages of Ni–Co–Se/CNT including homogeneous dispersion of Ni–Co–Se nanoparticles and interconnected networks morphology. The excellent electrical and mechanical properties of CNTs can not only improve the electrode conductivity but also well buffer the large volume changes during cycling. The above results show that Ni–Co–Se/CNT composite exhibits outstanding Li-ion storage performance with respect to high reversible Li-storage capacity, high cyclability and high rate performance for Li-ion batteries.

## 4. Conclusions

In summary, well dispersed Ni–Co–Se nanoparticles-MWNTs blended anode materials have been prepared for LIBs. The characterization of the materials demonstrated that the Ni–Co–Se nanoparticles of several nanometers in size were tightly anchored on a CNT conductive backbone network, which contributed to the enhancement of overall electronic conductivity as well as Li^+^ diffusivity. At a current density of 0.5 A g^−1^, the Ni–Co–Se/CNT can still exhibit a 687.8 mA h g^−1^ capacity after 100 charge–discharge cycles. The synergistic effect of Ni and Co ions and the formed network structure, result in a superior cycling stability. Therefore, it is anticipated that the Ni–Co–Se/CNT composite will have promising potential for application in high-performance LIBs.

## Conflicts of interest

The authors declare no conflict of interest.

## Supplementary Material

RA-008-C8RA05142A-s001

## References

[cit1] Tarascon J. M., Armand M. (2001). Nature.

[cit2] Magasinski A., Dixon P., Hertzberg B., Kvit A., Ayala J., Yushin G. (2010). Nat. Mater..

[cit3] Idota Y., Kubota T., Matsufuji A., Maekawa Y., Miyasaka T. (1997). Science.

[cit4] Wang H., Xi L., Tucek J., Ma C., Yang G., Leung M. K. H., Zboril R., Niu C., Rogach A. L. (2014). ChemElectroChem.

[cit5] Wang H., Wu Q., Cao D., Lu X., Wang J., Leung M. K. H., Cheng S., Lu L., Niu C. (2016). Materials Today Energy.

[cit6] Hou J., Shao Y., Ellis M. W., Moore R. B., Yi B. (2011). Phys. Chem. Chem. Phys..

[cit7] Persson K., Sethuraman V. A., Hardwick L. J., Hinuma Y., Ying S. M., Ven A. V. D., Srinivasan V., Kostecki R., Ceder G. (2010). J. Phys. Chem. Lett..

[cit8] Wu R., Wang D. P., Rui X., Liu B., Zhou K., Law A. W., Yan Q., Wei J., Chen Z. (2015). Adv. Mater..

[cit9] Choi S. H., Kang Y. C. (2014). Small.

[cit10] Yu X. Y., Hu H., Wang Y., Chen H., Lou X. W. (2015). Angew. Chem., Int. Ed..

[cit11] Zhu C., Mu X., Aken P. A. V., Yu Y., Maier J. (2014). Angew. Chem., Int. Ed..

[cit12] Wang Z., Liang Z., Xiong W. L. (2012). Adv. Mater..

[cit13] Hu H., Yu L., Gao X., Lin Z., Xiong W. L. (2015). Energy Environ. Sci..

[cit14] Park G. D., Kim J. H., Yun C. K. (2016). Mater. Charact..

[cit15] Zhang Z., Shi X., Yang X. (2016). Electrochim. Acta.

[cit16] He J., Chen Y., Li P., Fu F., Wang Z., Zhang W. (2015). Electrochim. Acta.

[cit17] Xie X., Ao Z., Su D., Zhang J., Wang G. (2015). Adv. Funct. Mater..

[cit18] Chen Y. M., Yu X. Y., Li Z., Ungyu P., Lou X. W. (2016). Sci. Adv..

[cit19] Ding S., Chen J. S., Lou X. W. (2011). Chem.–Eur. J..

[cit20] Xu C., Sun J., Gao L. (2011). J. Power Sources.

[cit21] Wang Z., Luan D., Madhavi S., Hu Y., Lou X. W. (2012). Energy Environ. Sci..

[cit22] Qin J., Zhang Q., Cao Z., Li X., Hu C., Wei B. (2013). Nano Energy.

[cit23] Wen Z., Wang Q., Zhang Q., Li J. (2010). Adv. Funct. Mater..

[cit24] Azadehranjbar S., Karimzadeh F., Enayati M. H. (2012). Adv. Powder Technol..

[cit25] Kong D., Wang H., Lu Z., Cui Y. (2014). J. Am. Chem. Soc..

[cit26] Lee C. T., Peng J. D., Li C. T., Tsai Y. L., Vittal R., Ho K. C. (2014). Nano Energy.

[cit27] Xiong X., Waller G., Ding D., Chen D., Rainwater B., Zhao B., Wang Z., Liu M. (2015). Nano Energy.

[cit28] Wei W., Mi L., Gao Y., Zheng Z., Chen W., Guan X. (2014). Chem. Mater..

[cit29] Chen H., Jiang J., Zhao Y., Zhang L., Guo D., Xia D. (2014). J. Mater. Chem. A.

[cit30] Liu B., Kong D., Zhang J., Wang Y., Chen T., Cheng C., Yang H. Y. (2016). J. Mater. Chem. A.

[cit31] Kwak I. H., Im H. S., Jang D. M., Kim Y. W., Park K., Lim Y. R., Cha E. H., Park J. (2016). ACS Appl. Mater. Interfaces.

[cit32] Li J. Y. D., Lu T., Yao Y., Pan L. (2017). Chem. Eng. J..

[cit33] Hu H., Zhang J., Guan B., Lou X. W. (2016). Angew. Chem., Int. Ed..

