# Supplementary material for: Facile hydrothermal synthesis of ternary Ni–Co–Se/carbon nanotube nanocomposites as advanced electrodes for lithium storage
Source: RSC Adv. 2018 Aug 13;8(50):28710–5. doi: 10.1039/c8ra05142a (PMC9084330; doi:10.1039/c8ra05142a)
Supplement: RA-008-C8RA05142A-s001 [file RA-008-C8RA05142A-s001.pdf]

Electronic Supplementary Material (ESI) for RSC Advances.
This journal is © The Royal Society of Chemistry 2018

Supporting Information

Jingguo Ding, Hui Xu, Xiaobo Chen*

School of New Energy and Electronic Engineering, Yancheng Teachers University, Yancheng, 224051, PR China

(*) Corresponding autor: E-mail: chenxbok@126.com, Tel: 86-515-88233177

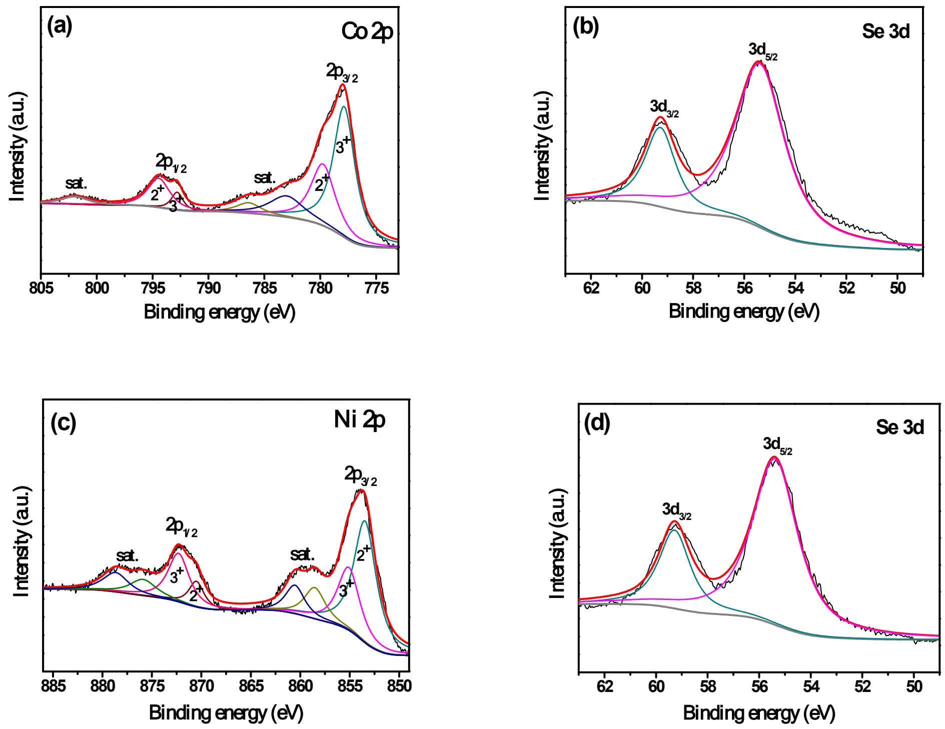

Fig.S1.high resolution XPS spectra:(a) Co 2p and (b) Se 3d of $CoSe_2$/CNT, (c) Co 2p and (d) Se 3d of $NiSe_2$/CNT.

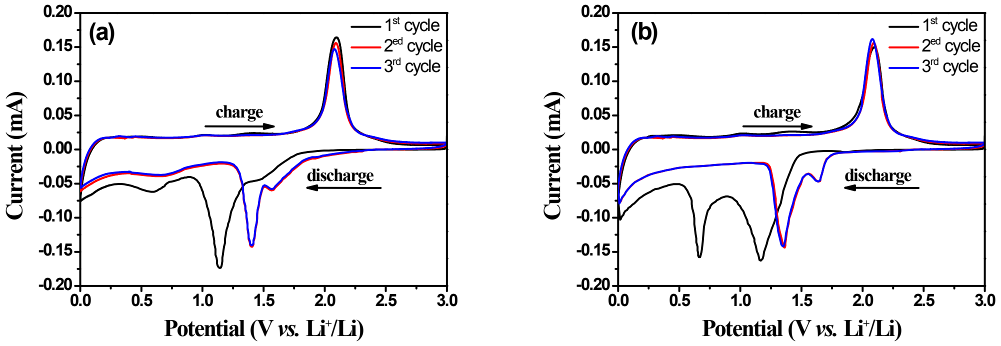

Fig.S2. Representative CV curves of (a) $CoSe_2$/CNTand (b) $NiSe_2$/CNT.

Table S1 Electrochemical performances comparison of this work versus the reported transition-metal chalcogenide based anode materials for LIBs.

Materials	Current density	Cycles	Capacity (mAh/g)	Reference
Ni-Co-Se/CNT	0.5 A/g	100	687.8	This work
CoSe@PCP	0.2 A/g	100	675	1
$Co_{0.85}Se$ Nanosheet	0.1 A/g	50	516	2
CoSe@C Hollow box	0.2 A/g	100	860	3
NC/CoS_2-650	0.1 A/g	50	560	4
Worm-like CoS_2	0.1 A/g	100	883	5
Yolk-shell CoS_2@NG	0.1 A/g	150	621	6
Ni_3Se_2	0.5 A/g	100	626.7	7
NiSe/C Core-shell sphere	0.1 A/g	50	428	8
$NiSe_2$/C Nanoplate	1 A/g	100	428	9
NiSe Thin film	0.1 A/g	200	314.9	10
NiS_2@CoS_2@C@C nanocubes	0.1 A/g	100	680	11
$MoSe_2$/C Sheet	0.1 A/g	100	150	12
$MoSe_2$@C Hollow sphere	1 A/g	100	681	13
$MoSe_2$/rGO Nanosheet	0.1 A/g	100	715	14
VSe/graphene	0.1 A/g	60	632	15

References

[1] J. Li, D. Yan, T. Lu, et al. An advanced CoSe embedded within porous carbon polyhedra hybrid for high performance lithium-ion and sodium-ion batteries. Chem. Eng. J., 2017, 325: 14-24.

[2] J.S. Zhou, Y. Wang, J. Zhang, T.P. Chen, H.H. Song and H. Y. Yang. Two dimensional layered $Co_{0.85}Se$ nanosheets as a high-capacity anode for lithium-ion batteries. Nanoscale, 2016, 8, 14992-15000.

[3] H. Hu, J.T. Zhang, B.Y. Guan, and X. W. (David) Lou. Unusual Formation of

CoSe@carbon Nanoboxes, which have an Inhomogeneous Shell, for Efficient Lithium Storage. Angew. Chem. Int. Ed. 2016, 55, 9514-9518.

[4] Q. Wang, R. Zou, W. Xia, et al. Facile Synthesis of Ultrasmall CoS2 Nanoparticles within Thin N‐Doped Porous Carbon Shell for High Performance Lithium‐Ion Batteries. Small, 2015, 11(21): 2511-2517..

[5] R. Jin, L. Yang, G. Li, et al. Hierarchical worm-like CoS 2 composed of ultrathin nanosheets as an anode material for lithium-ion batteries. J. Mater. Chem. A, 2015, 3(20): 10677-10680.

[6] W. Qiu, J. Jiao, J. Xia, et al. A Self‐Standing and Flexible Electrode of Yolk–Shell CoS2 Spheres Encapsulated with Nitrogen‐Doped Graphene for High‐Performance Lithium‐Ion Batteries. Chem.-Eur. J., 2015, 21(11): 4359-4367.

[7] X. Li, S. Li, Z. Zhang, et al. Ni 3 Se 2, electrodes for high performance lithium-ion and sodium-ion batteries. Mater. Lett., 2018, 220:86–89.

[8] Z.A. Zhang, X.D. Shi, X. Yang. Synthesis of core-shell NiSe/C nanospheres as anodes for lithium and sodium storage. Electrochim. Acta, 2016, 208, 238-243.

[9] H.S. Fan, H. Yu, X.L. Wu, Y. Zhang, Z.Z. Luo, H.W. Wang, Y.Y. Guo, S.Madhavi, and Q.Y. Yan. Controllable Preparation of Square Nickel Chalcogenide (NiS and $NiSe_2$) Nanoplates for Superior Li/Na Ion Storage Properties. ACS Appl. Mater. Interfaces, 2016, 8, 25261-25267.

[10] M.Z. Xue, Z.W. Fu. Lithium electrochemistry of $NiSe_2$: A new kind of storage energy material. Electrochem. Comm. 2006, 8, 1855-1862.

[11] Y. Lin, Z. Qiu, D. Li, et al. NiS 2 @CoS 2, nanocrystals encapsulated in N-doped carbon nanocubes for high performance lithium/sodium ion batteries. Energy Storage Mater., 2017.

[12] Y. Liu, M.Q. Zhu and D. Chen. Sheet-like $MoSe_2$/C composites with enhanced Li ion storage properties. J. Mater. Chem. A, 2015, 3, 11857-11862.

[13] X. Yang, Z.A. Zhang, Y. Fu and Q. Li. Porous hollow carbon spheres decorated with molybdenum diselenide nanosheets as anodes for highly reversible lithium and sodium storage. Nanoscale, 2015, 7, 10198-10203.

[14] Z.G. Luo, J. Zhou, L.R. Wang, G.Z. Fang, A.Q. Pan and S.Q. Liang. Two-dimensional hybrid nanosheets of few layered $MoSe_2$ on reduced graphene oxide as anodes for long-cycle-life lithium-ion batteries. J. Mater. Chem. A, 2016, 4, 15302-15308.

[15] Y. Wang, B. Qian, H. Li, L. Liu, L. Chen, H. Jiang, VSe2/graphene nanocomposites as anode materials for lithium-ion batteries, Mater. Lett. 141 (2015) 35-38.
